# The Latest Exploration of Cerebrovascular Diseases: From Preclinical Research to Treatment

**DOI:** 10.3390/brainsci15121281

**Published:** 2025-11-28

**Authors:** Basil Erwin Grüter, Martina Sebök

**Affiliations:** 1 Department of Neurosurgery and Neuroradiology, HOCH Health Ostschweiz, 9000 St. Gallen, Switzerland; 2 Department of Neurosurgery, Clinical Neuroscience Center, University Hospital Zurich, University of Zurich, 8091 Zurich, Switzerland; martina.seboek@usz.ch

## 1. Introduction

Cerebrovascular diseases represent one of the most significant challenges in modern clinical neuroscience, encompassing a spectrum of disorders in which disturbances of cerebral blood flow, vascular integrity, and hemodynamic regulation culminate in devastating neurological sequelae [1,2]. Recognizing the complex interplay of these mechanisms, this *Special Issue* was conceived to capture cutting-edge research spanning mechanistic and preclinical investigations through to translational and clinical treatment studies. Particularly noteworthy are the rapid advances in imaging technologies, biomarker discovery, endovascular and microsurgical techniques, regenerative and neuroprotective strategies, and, more recently, the integration of artificial intelligence into cerebrovascular diagnostics and therapeutic planning.

In this Editorial, we provide an overview of the works published within this Special Issue, delineate persisting gaps in our understanding, and propose future research directions to guide the field toward more individualized and effective cerebrovascular care.

## 2. Overview of Published Works

The Special Issue brings together twenty articles that collectively map the broad continuum of cerebrovascular research—from computational modeling of aneurysm formation to clinical studies on complex vascular malformations, and from the refinement of imaging biomarkers to the development of novel drug delivery technologies. The publications can be grouped into four overarching research domains:

1. Mechanistic simulations and modeling studies;
2. Imaging and diagnostic biomarker investigations;
3. Interventional and therapeutic innovations;
4. Artificial Intelligence and decision-support systems.

Mechanistic studies have provided crucial insights into the pathophysiological processes underlying cerebrovascular disease, laying the foundation for improved clinical comprehension [3]. Advances in imaging and the development of vessel-wall biomarkers have expanded the diagnostic repertoire, improving both detection and risk stratification [4]. Large surgical and endovascular cohorts have offered valuable real-world data on treatment outcomes and procedural strategies, supporting the refinement of evidence-based decision-making.

At the therapeutic frontier, innovative delivery systems—such as ultrasound-mediated nimodipine release—have illustrated promising alternatives beyond conventional care paradigms. Meanwhile, the first explorations into the role of artificial intelligence and large language models in clinical decision analysis have signaled a future in which computational tools may augment, rather than replace, clinician judgment.

However, significant challenges persist. Mechanistic models, though increasingly sophisticated, remain distant from clinical integration. Imaging biomarkers require prospective validation in large, heterogeneous populations. Many treatment strategies continue to rely on expert consensus rather than robust randomized evidence, particularly for rare vascular malformations. Artificial intelligence applications remain preliminary, often limited to retrospective analysis without demonstrated impact on patient outcomes. Similarly, drug delivery systems and nanocarriers, while conceptually elegant, have yet to advance beyond preclinical evaluation [5].

## 3. Focus on Future Research Directions

Building on the collective contributions of this Special Issue, several key avenues for future research can be delineated:

**1. Bridging mechanistic modeling and individualized risk prediction**

Fluid–structure interaction studies of aneurysm initiation and modeling approaches in leukoaraiosis underscore the value of mechanistic insight. The next step lies in translating these models into patient-specific risk assessment tools—integrating hemodynamic, geometric, and histopathological parameters to form individualized treatment strategies [6].

**2. Translating novel delivery and regenerative technologies**

The development of nimodipine nanocarriers exemplifies the potential for controlled, on-demand pharmacologic interventions targeting subarachnoid hemorrhage, vasospasm, or focal ischemia [7]. To move toward clinical application, forthcoming studies should prioritize in vivo validation, toxicological safety profiling, and early-phase clinical trials, ideally within multidisciplinary translational frameworks.

**3. Addressing venous and microvascular pathophysiology**

While arterial mechanisms have dominated research, venous outflow resistance and microvascular dysfunction—implicated in entities such as leukoaraiosis—remain underexplored. Systematic investigations into the venous compartment, microcirculatory flow, and neurovascular unit integrity will be crucial to comprehensively understanding cerebrovascular disease [8].

**4. Prospective validation of imaging biomarkers**

Imaging markers, such as vessel-wall contrast enhancement in Moyamoya disease and intracranial aneurysms, have demonstrated potential as predictive tools. Prospective multicenter validation and integration into structured clinical algorithms are essential. Analogous biomarker development should extend to other pathologies, including AVMs and perforator aneurysms, to enable earlier and more stratified interventions [9].

**5. Responsible integration of artificial intelligence**

Initial evidence indicates that AI models can approximate multidisciplinary board decisions for unruptured intracranial aneurysms. Future research must evaluate real-world deployment, focusing on safety, interpretability, clinician acceptance, and measurable impact on outcomes [10]. Ethical oversight and medico-legal governance must evolve concurrently to ensure responsible adoption.

**6. Advancing precision medicine and molecular targeting**

The movement toward precision medicine will increasingly rely on genetic, hemodynamic, and anatomical phenotyping. In AVMs, for instance, emerging data on somatic KRAS and BRAF mutations suggest the feasibility of targeted molecular interventions [11]. Broader incorporation of molecular diagnostics into cerebrovascular management is likely to define the next decade of translational research.

**7. Strengthening evidence through multicenter clinical trials**

While large retrospective cohorts remain informative, randomized and multicenter prospective studies are urgently needed to refine decision-making, particularly for rare lesions such as AVMs, combined aneurysm–stenosis cases and surgical treatment of (acute) stroke [12,13]. Such studies will form the backbone of evidence-based cerebrovascular therapy.

## 4. Conclusions

The Special Issue “*The Latest Exploration of Cerebrovascular Diseases: From Preclinical Research to Treatment*” encapsulates the dynamic evolution of the field—from hemodynamic modeling to advanced imaging, from clinical cohorts to interventional innovation, and from artificial intelligence to precision nanomedicine. Yet, the translation of mechanistic insights into clinical applicability, the validation of biomarkers, the conduction of high-quality interventional trials, and the ethical integration of AI remain critical frontiers.

We invite the readership—clinicians, scientists, bioengineers, and data specialists alike—to engage with the comprehensive body of work presented herein, to build upon its foundations, and to contribute toward the next generation of cerebrovascular research. The convergence of mechanistic, imaging, therapeutic, and computational advances heralds a transformative era in cerebrovascular medicine—one defined by precision, personalization, and improved patient outcomes.

In this spirit, we already look forward to the second volume of the Special Issue, which will continue this trajectory toward bridging experimental insight and clinical translation in the management of cerebrovascular disease.
